# Comparative Study on the Efficiency of the Photodynamic Inactivation of *Candida albicans* Using CdTe Quantum Dots, Zn(II) Porphyrin and Their Conjugates as Photosensitizers

**DOI:** 10.3390/molecules20058893

**Published:** 2015-05-18

**Authors:** Osnir S. Viana, Martha S. Ribeiro, Andréa C. D. Rodas, Júlio S. Rebouças, Adriana Fontes, Beate S. Santos

**Affiliations:** 1 Pharmaceutical Sciences Department, Pernambuco Federal University, Recife 50670-901, Brazil; E-Mail: osnirviana@yahoo.com.br; 2 Center for Lasers and Applications, IPEN-CNEN-SP, São Paulo 05508-000, Brazil; E-Mails: marthasr@usp.br (M.S.R.); andrea.ipen@gmail.com (A.C.D.R.); 3 Chemistry Department, CCEN Universidade Federal da Paraiba, João Pessoa 58051-900, Brazil; E-Mail: jsreboucas@quimica.ufpb.br; 4 Biophysics and Radiobiology Department, Pernambuco Federal University, Recife 50670-901, Brazil; E-Mail: adriana.fontes.biofisica@gmail.com

**Keywords:** quantum dots, Zn-porphyrins, photodynamic therapy, photodynamic inactivation, *Candida albicans*, fibroblasts

## Abstract

The application of fluorescent II-VI semiconductor quantum dots (QDs) as active photosensitizers in photodymanic inactivation (PDI) is still being evaluated. In the present study, we prepared 3 nm size CdTe QDs coated with mercaptosuccinic acid and conjugated them electrostatically with Zn(II) *meso*-tetrakis (*N*-ethyl-2-pyridinium-2-yl) porphyrin (ZnTE-2-PyP or ZnP), thus producing QDs-ZnP conjugates. We evaluated the capability of the systems, bare QDs and conjugates, to produce reactive oxygen species (ROS) and applied them in photodynamic inactivation in cultures of *Candida albicans* by irradiating the QDs and testing the hypothesis of a possible combined contribution of the PDI action. Tests of *in vitro* cytotoxicity and phototoxicity in fibroblasts were also performed in the presence and absence of light irradiation. The overall results showed an efficient ROS production for all tested systems and a low cytotoxicity (cell viability >90%) in the absence of radiation. Fibroblasts incubated with the QDs-ZnP and subjected to irradiation showed a higher cytotoxicity (cell viability <90%) depending on QD concentration compared to the bare groups. The PDI effects of bare CdTe QD on *Candida albicans* demonstrated a lower reduction of the cell viability (~1 log10) compared to bare ZnP which showed a high microbicidal activity (~3 log10) when photoactivated. The QD-ZnP conjugates also showed reduced photodynamic activity against *C. albicans* compared to bare ZnP and we suggest that the conjugation with QDs prevents the transmembrane cellular uptake of the ZnP molecules, reducing their photoactivity.

## 1. Introduction

Photodynamic therapy (PDT) is a relatively new type of therapy used for the treatment of tumorous cells/tissues and pathogenic microorganisms [1]. For antibacterial treatments the term Photodynamic Inactivation (PDI) is also frequently applied. Some types of cancer and local infections have been treated with PDT, by applying a photosensitizing substance (PS) followed by photoaactivation. The basic principle of photodynamic therapy is that a photoactive PS drug causes the transfer of an electron or the transfer of energy to molecular oxygen generating reactive oxygen intermediaries (ROI) or *reactive oxygen species* (ROS), which immediately react with biomolecules in cell organelles, causing damage and resulting in cell death [2,3]. The main mechanism of PDI is thus related to the formation of free radicals, such as the hydroxyl radical (^•^OH), the superoxide radical (O_2_^•−^) or non-radical species, such as singlet oxygen (^1^O_2_) and hydrogen peroxide (H_2_O_2_) [4,5,6,7]. There are various factors that influence the cell death, including PS type, concentration, incubation time, the availability of oxygen and also the wavelength, the power density (mW·cm^−2^) and light energy dose (J·cm^−2^) of the irradiation source [8].

Among classical photosensitizing drugs that are now commercially available there are the porphyrins, which have been applied clinically in photodynamic therapy and photodynamic inactivation for various types of cancer and microorganisms [9,10,11,12] due to their high level of efficiency in terms of ROS production [13]. Porphyrins are cyclic organic molecules containing four nitrogen atoms arranged in a macrocyclic structure called a tetrapyrrole. These highly conjugated structures and their closed-shell complex counterparts, such as Zn(II) porphyrins, are highly fluorescent compounds largely studied as efficient photosensitizing agents [14,15]. Cationic Zn(II) complexes derived from *N*-alkylpyridylporphyrins are promising photosensitizing agents, capable of binding to cell membranes and targeting sub-cellular components, such as mitochondria. The mechanism of action of these compounds has often associated their high uptake and light-dependent ROS production to the resulting photodamage to lipid membranes and respiratory mitochondrial complexes [13,14,16,17,18,19,20,21].

Quantum dots (QDs) have emerged as an alternative to traditional PDI drugs, either bare or associated to other photosensitizing compounds [22,23,24,25]. Quantum dots are semiconductor nanocrystals composed of binary or ternary combinations from elements of the II–VI or III–V families of the Periodic Table. They possess very interesting electrical and optical properties and are currently used in various fields ranging from active opto-electrical devices, sensors and more recently as a new class of fluorescent biological probes. The luminescent properties of QDs make them able to label specific cells according to their surface functionality and they have shown a better performance when compared with commercially available organic dyes, due to the higher degree of photostability [26,27,28]. In addition to presenting exceptionally stable photoluminescence in relation to conventional fluorophores, QDs have a high quantum yield, which strengthens their application potential to stain cells and molecules in various types of tissues [6,21,29,30,31,32].

**Figure 1:**
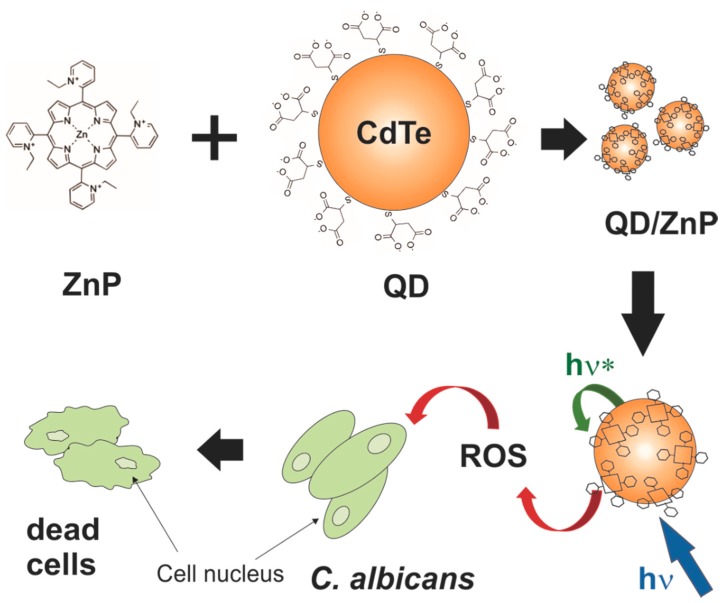
Schematic representation of the hypothesis tested here showing the cationic Zn(II)-*meso*-tetrakis(*N-*ethylpyridinium-2-yl)porphyrin (ZnP) conjugated to mercaptosuccinic acetate-stabilized CdTe QDs. The purple arrows (hν) represent the light excitation of the QDs while the blue arrow (hν*) represents the energy transfer pathway from the QDs to the conjugated ZnP species. The excitation of this system may induce the formation of reactive oxygen species (ROS) that applied to *C. albicans* cells would induce their death.

In the last decade different research groups have been investigating the use of this new class of fluorophores as new photosensitizers in PDI. These nanostructures show not only efficient emitting light capability within the biological environment, but also the possibility, when photoexcited, of transferring charge to the oxygen molecules nearby inducing ROS. Since 2000, semiconductor QDs have been tested for ROS production and by now several advantages over the organic PS may be pointed out [2]: (i) a broader absorption band, enabling a more convenient excitation process; (ii) large extinction coefficients (from 10^5^ to 10^6^ M^−1^cm^−1^ at first excitonic band); (iii) narrow emission bands (bandwidth ~25–50 nm) with high quantum yield; (iv) a chemically active surface allowing therapeutic targeting and (v) a very high photostability. This last optical property is especially appropriate for longer irradiation periods. Based on these properties quantum dots alone or in combination with conventional PS are being tested [33,34,35,36,37] in PDI. The association of QDs and porphyrins may result in alternative PS candidates in the search for a more powerful generation of ROS for photodynamic therapy against cancer cells and pathogenic microorganisms. Among pathogens of clinical importance *Candida albicans* is worth highlighting due to its notable virulence. *Candida albicans* is recognized as the most common fungal pathogens causing superficial infections of skin and mucosal membranes, reaching systemic levels, especially in immunocompromised individuals [38,39]. The resistance of *C. albicans* strains against classical antifungals such as fluconazole has increased, which drives the search for new therapeutic alternatives [40,41,42]. Porphyrin and non-porphyrin mediated photodynamic inactivation has already been applied successfully for some types of microorganisms, including *Candida albicans* [12,16,43,44,45,46,47,48], but its association to QDs as new PS candidates in PDI experiments is still being tested and almost all of the systems consist of lipophilic porphyrins or hydrophobic QDs. In the present study, we carried out the synthesis of water dispersed CdTe QDs and their conjugation to a highly hydrophilic cationic Zn(II) porphyrin, *ortho* Zn(II) *meso*-tetrakis(*N-*ethylpyridinium-2-yl)porphyrin [ZnTE-2-PyP^4+^ (abbreviated here as ZnP)], and evaluated their potential, either free or conjugated, in the photodynamic action against *Candida albicans* yeast cells*.* ZnP (Figure 1) is a cationic hydrophilic porphyrin that has shown a very good photodynamic activity against Gram-negative bacteria and cancer cells [16]. The main idea of this study (represented in Figure 1) was to verify the ability of the QDs to transfer energy to the porphyrin when irradiated and to cause more effective cell damage through PDI action. This class of porphyrins presents emission bands in the red region allowing its PDI action in a deeper tissue profile.

## 2. Results and Discussion

### 2.1. Structural and Optical Characterization of the QDs, ZnP and QDs-ZnP

**Figure 2:**
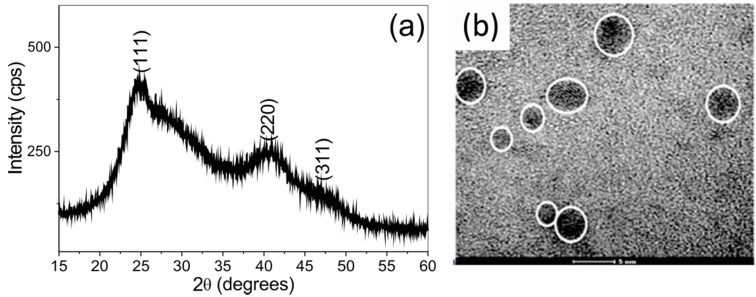
Structural characterization of the CdTe QDs used here. (**a**) A representative X-ray powder diffratogram of CdTe-MSA dried nanocrystals; (**b**) Transmission electron microscopy of CdTe-MSA. The circled electron dense structures represent individual nanoparticles. Bar = 5 nm.

The powder diffraction pattern of dried QD samples (Figure 2a) shows the characteristic CdTe crystalline plane profile thickened by the nanoscale dimension of the particles. Data from the *Joint Committee on Powder Diffraction Standards* crystallographic library [49] strongly suggest that the peaks located at 24.7; 39.0 and 46.6 degrees (2θ) correspond, respectively, to the (111), (220) and (311) crystallographic planes of CdTe possessing the zinc blend cubic structure. 

**Figure 3:**
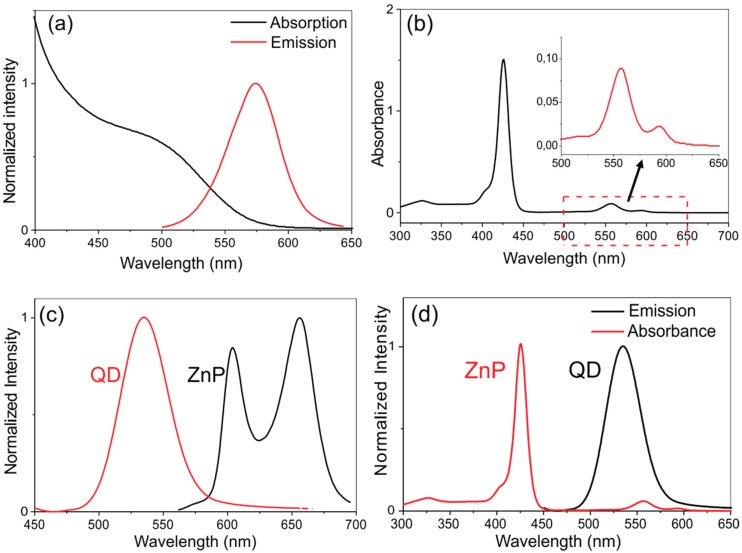
(**a**) Absorption and emission spectra of CdTe-MSA QDs (Exc: 460 nm); (**b**) absorption spectrum for the ZnP (the insert amplified the 500–650 nm spectral range); (**c**) superposition of the normalized emission spectra of CdTe QDs and the ZnP solution; (**d**) normalized absorption spectrum of ZnP and emission spectrum of CdTe QDs (λ_exc_ = 460 nm).

By applying Scherrer’s equation we estimate a mean particle size of *d* = 3.1 nm. This estimation is corroborated by the TEM analysis, which shows particles within the 2–5 nm size range (Figure 2b). The optical properties of the synthesized QDs are presented in Figure 3a. The absorption spectra of the synthesized CdTe QDs show the same features as other similar CdTe systems described in the literature [34,50,51,52] and allowed the estimation of the average particle size by applying Yu´s semi-empirical equation (Equation (1)) [53]:

*d* = (9.8127 × 10^−7^) λ^3^ − (1.7147 × 10^−3^) λ^2^ + (1.0064) λ −194.84 (1)

where *d* is the mean QD diameter and λ is the maximum wavelength in nm corresponding to the first excitonic absorption peak.

Observing the first absorption maximum at λ = 505 nm we estimated an average size of *d* = 2.7 nm for the CdTe-MSA QDs which is in good agreement to the structural size estimation. Figure 3a also shows the emission band of the CdTe-MSA nanocrystals with maximum at λ = 575 nm (λ_exc_ = 460 nm) and a bandwidth (full width at half maximum—FWHM) of 42 nm. This emission band corresponds to the S-S exciton recombination of the CdTe nanoparticles. The characteristic absorption and emission spectra of the ZnP are presented in Figure 3b,c, respectively. Figure 3d shows an overlap of the emission spectrum of the QDs and the absorption spectrum of the ZnP porphyrin.

The absorption spectrum of ZnP is marked by an intense absorption band (the *Soret* band) at λ = 420 nm along with two bands (Q bands) with intensity maxima at 560 and 590 nm, in agreement with data reported by Benov *et al.* [17]. The emission spectra of ZnP show two intense bands in the red region (λ = 596 and 657 nm) with excitation at λ = 420 nm, consistent with the spectroscopic features reported for the analogous methyl complex [54]. Analyzing the spectra profiles presented by both species (Figure 3a,d) we note that in order to test the hypothesis of energy transfer from the QDs to the ZnP molecules, the systems must be excited next to the first maximum of the absorption band of the QDs and in a spectral region where no direct absorption of the porphyrin is expected. The chosen wavelength to study the PDI action of these systems was therefore λ = 460 nm.

The surface charges of the nanoparticles as inferred by their zeta potential (ζ = −57 mV) indicate that, as expected, the CdTe-MSA particles are negative. The addition of ZnP molecules to CdTe-MSA QDs suspensions kept in a fixed pH ~7 is followed by an decrease in modulus of the zeta potential in a concentration-dependent fashion (ζ = −42, −36 and −27 mV were observed for 1.5:0.15; 1.5:0.25 and 1.5:0.5 QDs:ZnP ratio respectively), strongly suggesting that the positive macromolecules are conjugated to the negative surface of the CdTe-MSA nanocrystals, most probably by adsorption.

### 2.2. Spectroscopic Studies of the QD-ZnP Conjugates

**Figure 4:**
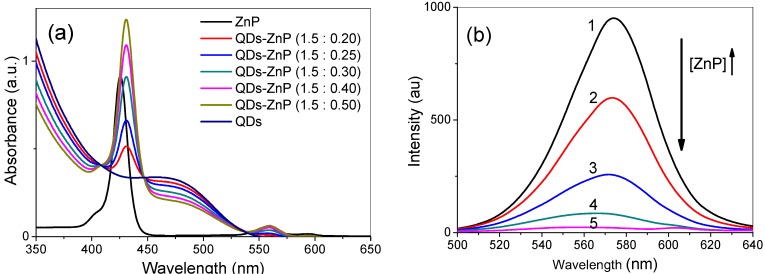
(**a**) Absorption spectra of different molar ratios of QDs:ZnP; (**b**) Emission spectra of CdTe-MSA QDs (λ_exc_ = 460 nm) with addition of increasing quantities of ZnP (1) = QDs; (2) QDs:ZnP 1:0.12; (3) QDs:ZnP = 1:0.25; (4) QDs:ZnP = 1:0.5; (5) QDs:ZnP = 1:1.

Before conducting the assays related to the production of ROS and anti-*Candida* PDI, the stability, as well as the spectroscopic changes of the conjugates containing different QD:ZnP ratios, were evaluated using their corresponding absorption and emission spectra. No evidence of precipitation or other incompatibility of the conjugated systems was observed, even after 12 weeks of storage. Moreover, the individual characteristic profiles of the absorption spectra of free QDs and ZnP were maintained upon their mixture at different ratios (Figure 4b) and reflect the overlap of their individual spectra. 

On the other hand, the emission spectrum intensity of the QDs gradually decreased with increasing amount of conjugated ZnP molecules (Figure 4b). The luminescence quenching of QDs upon increasing the net concentration of porphyrins was reported in the literature and the following processes were applied to explain this effect: (i) dynamic or static energy transfer between the QDs and the photosensitizer triggered by radiative energy transfer or Förster Resonance Energy Transference (FRET) [55,56,57,58] and (ii) competition among the absorbers [50]. As ZnP molecules present practically no absorbance at the excitation wavelength applied (460 nm) we rule out the competition hypothesis and we suggest that there is a high probability of energy transfer from the QDs to the ZnP molecules.

### 2.3. Indirect Detection of ROS

**Figure 5:**
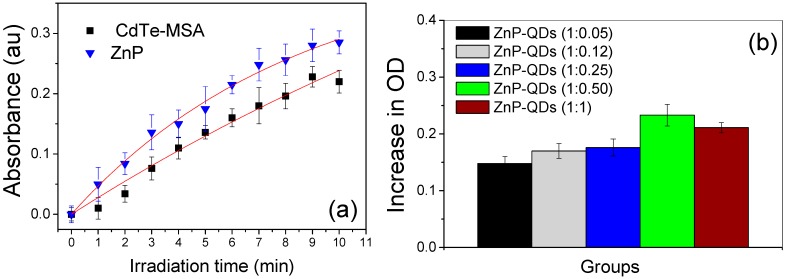
Indirect detection of ROS generation monitored by the increase in optical density (OD) at 580 nm (NBT oxidation). (**a**) Generation of ROS upon irradiation of ZnP (blue triangles, R^2^ = 0.997) and CdTe-MSA QDs (black squares, R^2^ = 0.965 ([PS] = 1 µmol·L^−1^) and (**b**) ROS generation upon irradiation for 10 min of mixtures containing different proportions of ZnP and CdTe-MSA QDs. Analysis of variance (ANOVA) showed that the differences among the various molar fractions of QDs-ZnP conjugates were significant (*p* < 0.01).

One of the main mechanisms for evaluating the potential of a certain photosensitizer-candidate for photodynamic therapy is to measure its capacity for generating singlet oxygen, superoxide and other reactive oxygen species [25,59,60]. Here we tested the efficiency of this mechanism by indirectly detecting the ROS production by oxidation of NBT molecules in the presence of NADH. The graphs in Figure 5 show the dynamics of the formation of reactive oxygen species via irradiation of the photosensitizers (QDs, ZnP and QDs-ZnP) using this method. The oxidation of NBT produces an increase in the optical density of the sample at 580 nm, indicating the presence of ROS. The ZnP and QDs samples were tested individually as photosensitizers [Figure 5a—ROS production curve as a function of time (minutes)]. A greater rate of ROS production was found in the ZnP systems, which is consistent with the excellent photosensitizer behavior previously reported for this porphyrin [9,13,14,61]. To compare the rates of NBT oxidation, we assumed that the absorbance can be expressed by A(t) = A_0_(1 − αt), where A(t) is the intensity of absorbance at time, A_0_ is the minimum absorbance, and α is a fit parameter. Then, the rate of ROS generation can be calculated from the equation: V = dA(t)/dt [62]. Thus, the relative rate (V_ZnP_/V_CdTe-MSA_) of ROS formation of ZnP is approximately 27.5% faster than CdTe-MSA. 

At a constant molar concentration of ZnP, the addition of increasing amounts of CdTe-MSA QDs to yield QDs-ZnP conjugates of different molar composition was accompanied by an increase in ROS production up to a ZnP:QDs ratio of 1:0.5 (Figure 5b—10 min of irradiation). These results confirm the potential of QDs, ZnP, and conjugated QDs-ZnP to induce light-dependent ROS generation, although at a cellular level this effect may be strengthened or suppressed by interactions of the photosensitizers with cell components.

### 2.4. Cytotoxicity Assays of the PSs in Fibroblast Cells

**Figure 6:**
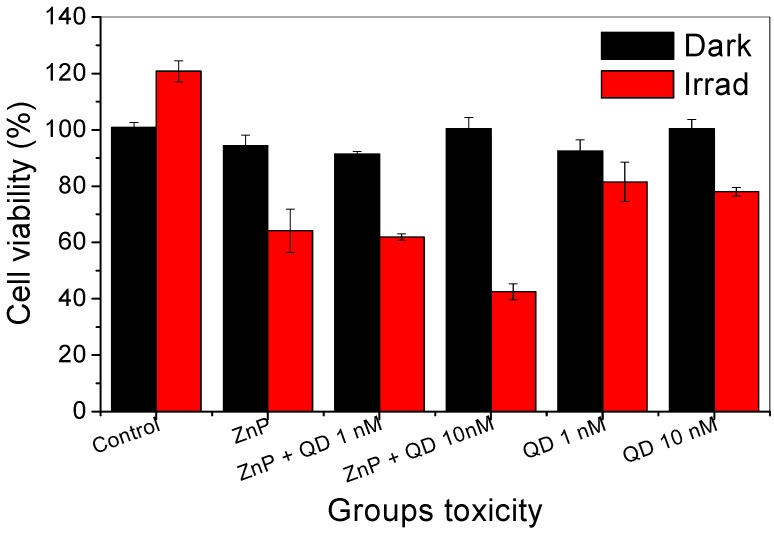
Measurement of cytotoxicity of fibroblasts cells treated with two different photosensitizers or their combinations: cells were exposed to (1) QDs (CdTe-MSA) in a concentration of 1 and 10 nmol·L^−1^; (2) ZnP kept at 10 µmol·L^−1^ and (3) QD/ZnP conjugates. All the systems were tested in the dark (dark shadow) or were irradiated at 460 nm (±20) 150 mW irradiation for 9 min (red shadow). The estimative of cytotoxicity was obtained by MTS test (data points show the mean standard deviation, *n* = 5). ANOVA results showed *p* < 0.05. All irradiated groups had a statistically significant difference compared to the non-irradiated control.

The PDI tests were preceded by fibroblast cytotoxicity assays in the dark and under irradiation, using the same range of concentrations that would be used for the other assays relating to the generation of ROS and PDI against *Candida albicans*. The results summarized in Figure 6 show that cells irradiated by blue light alone exhibited an increase of about 20% in cell viability. This result is not surprising since it is known that blue light irradiation regulates proliferation and differentiation in human skin cells depending on energy dose [63]. In fact, low levels of reactive oxygen species are generated during low energy visible light illumination of cells [64] and ROS are created following ligand-receptor interactions. They function as specific second messengers in signaling cascades involved in cell proliferation and differentiation [65] that may lead to cellular proliferation. Besides, the absorption spectrum of human fibroblast monolayers shows absorption peaks in the blue region of the electromagnetic spectrum. 

Fibroblasts incubated with the three types of photosensitizers for 10 min in the dark showed survival rates greater than 90%, showing that dark toxicity of the photosensitizers (ZnP, QDs and QDs-ZnP) was low. In the group of cells treated with QDs and then irradiated with 460 (±20) nm light, the cell survival rate was higher than or close to 80% at 1 to 10 nmol·L^−1^ concentration of QDs, which indicates that at this concentration range, QDs exerted low phototoxicity for these cells. The phototoxicity of ZnP studied individually or in combination with different concentrations of CdTe-MSA QDs was more pronounced than that observed with bare QDs. In fact, following irradiation, ZnP alone exhibited about 65% of cell viability. The QDs-ZnP conjugates produced a more significant decrease in cell viability ranging from 60% to 40% depending on increase of QD concentration.

### 2.5. Photodynamic Inactivation Assays for Candida albicans

**Figure 7:**
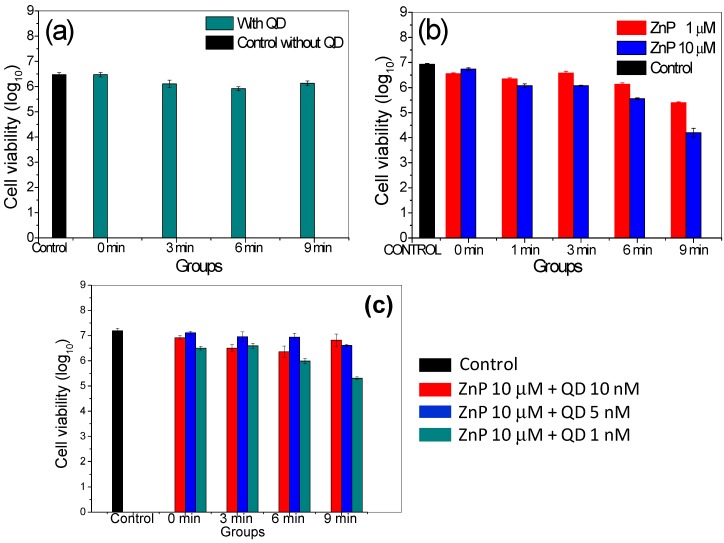
Evaluation of photodynamic inactivation of a culture of *Candida albicans* ATCC 10231. (**a**) Cell viability after addition of 20 µL of CdTe-MSA QD 10 nmol·L^−1^, under different irradiation doses with LED 460 nm (±20 nm). Between the control group and the non-irradiated QD group (0 min) there is no significant difference; there is a significant difference between the irradiated groups compared to the control group with *p* value < 0.01; (**b**) Cell viability after addition of 20 µL of ZnP 1 and 10 µmol·L^−1^, under different irradiation doses with LED 460 nm (±20 nm); there are statistically significant differences between all groups evaluated, with greater evidence for ZnP (10 µM) groups at times 6 and 9 min irradiation and ZnP (1 µM) in 9 min compared to the control group; (**c**) Cell viability after addition of 20 µL of different proportions of QDs-ZnP under different levels of irradiation. Control means non-irradiated cells. There are statistically significant differences between all groups evaluated, with greater evidence for QD-ZnP group (1 nM:10 µM and 9 min irradiation) compared to the control group. Control group (non-irradiated; incubation time = 9 min).

After observing the indirect light-dependent ROS production by QDs and ZnP photosensitizers, free or conjugated, and their effect on fibroblast cell cultures, these systems were tested as active components in antimicrobial photodynamic therapy. Owing to its medical importance and previous PDI background, *Candida albicans* was chosen as the model microorganism*.* The viability of these cells as a function of different exposure times is shown in Figure 7a for bare QDs system. According to the results, the cell viability of *C. albicans* decreased to a greater or lesser extent after the PDI session in the presence of the type of PS (QDs, ZnP, QDs-ZnP). It was especially noticeable that a low efficiency in PDI against *Candida albicans* occurred when applying QDs. Compared to the control groups, CdTe-MSA QDs barely altered *Candida* sp*.* cell viability (Figure 7a). These results demonstrate that, under the experimental conditions used here, the CdTe-MSA QDs show a low efficiency in the photodynamic inhibition of *Candida albicans*.

The use of Zn(II) porphyrin as photosensitizer against *C. albicans* resulted in a significant reduction of cell viability by 3 log units (Figure 7b), which reinforces its potential for use in antimicrobial photodynamic therapy, as also reported previously by Benov *et al*., who applied this porphyrin to *Escherichia coli* [17]. The positive charge of ZnP can speed up the absorption of the PS by the cell [14,61,66] and once internalized and photostimulated, ZnP could trigger internal cell apoptosis mechanisms [17]. It is worthwhile mentioning that all experiments were performed irradiating with a LED at 460 ± 20 nm which is not efficient for the porphyrin’s own excitation (Figure 3b). It is expected that its PS capability will be greatly enhanced using an irradiation wavelength at the maximum absorbance of the ZnP (*i.e*., 420 nm).

To evaluate the effect of photodynamic inhibition on the *C. albicans* cells by the QDs-ZnP conjugates, an assay was carried out preparing systems of different ratios, maintaining the concentration of porphyrin constant at 10 µmol·L^−1^ and varying the concentration of QDs. The results presented in Figure 7c show that there was no increase in the photodynamic effect on the microorganisms by conjugating QDs and ZnP and that the increase in the concentration of QDs had a negative impact on the production of ROS and consequently on cell death.

As described before, we tested the irradiation at 460 ± 20 nm to verify the hypothesis of a possible energy transfer mechanism from the QDs to the ZnP molecules in order to verify an overall increase in the PDI action. Interestingly, although barely any absorption of the ZnP is expected in this spectral range, its PDI action is greater than for the QDs alone and of the conjugate.

Energy transfer was suspected to be the main mechanism explaining the emission quenching of the QDs when the amount of ZnP molecules increases, as shown in Figure 4b, but this conclusion does not explain the PDI results. The PDI performance of the QDs and of the QDs-ZnP conjugates on C. *albicans* was almost negligible. In order to understand this result we have to remember that the overall PDI action takes into account the penetration of the PS within the cell and within the cell components. The cell distribution of ZnP and its analogues (*meta*- and *para*-ZnP, and similar Zn(II)*N*-alkylpyridylporphyrins) before and after light irradiation was already studied by some authors [16,17,18]. Ezzedine *et al*., showed that the methyl and ethyl ZnP porphyrins were dispersed in the cytosol and remained outside the nucleus, localizing predominantly in the lysosomes and that this distribution was concentration independent in the 5–20 µmol^−1^ range [18]. The PDI efficiency in cells depends highly on the PS cellular uptake by transmembrane channels and by endocytic processes. The conjugation of the ZnP molecules to the larger QDs (2–3 nm size) hinders an efficient transmembrane cellular uptake of the small porphyrin molecules (<1 nm). Fungal cells are comprised of a cell wall structure preventing the uptake of bare CdTe-MSA QDs [67], and consequently of QDs-ZnP conjugates, and we suggest that this decreased the ZnP PDI action. Further studies are needed to better understand these aspects.

In the last decade quantum dots have emerged as new PS candidates in PDI processes. Their tunable emission capability, allied to their surface activity and high photoresistance are listed as their great advantages for diverse fluorescence applications. Among several authors Samia *et al*., summarized their potential in PDI processes [24], mainly as energy donors to conventional PS through FRET mechanisms or in energy transfer processes that result in the production of ROS. Our present results point out that although isolated QDs present the capability of generating ROS, their application in biological environments or even in clinic trials is still an issue to be thoroughly studied. Their ability of interfering in the cell viability in PDI is also dependent on their cell uptake and retention time in the biological system, especially for cells that do not perform endocytosis. Thus, modifications of these systems, such as targeting the QDs to the cell membranes may guarantee their effective applications.

## 3. Experimental Section

### 3.1. Synthesis of CdTe QDs and ZnP

The synthesis of CdTe-MSA QDs was carried out according to a previously reported method [68], using the following reagents and solutions: cadmium perchlorate solution [Cd(ClO_4_)_2_] 0.01 mol·L^−1^, elementary tellurium (Te^0^), sodium borohydride (NaBH_4_) and mercaptosuccinic acid (MSA), all of them from Sigma-Aldrich (St Louis, MO, USA). The CdTe QDs were synthesized by mixing Cd(ClO_4_)_2_ (0.2 mmol) with the stabilizing agent (MSA, 0.46 mmol) in ultrapure water (40 mL). The pH of this mixture was adjusted according to the pKa of MSA and the mixture was heated to a constant temperature of 90 °C (±5 °C). In parallel, Te^2−^ ions were obtained by the NaBH_4_ induced reduction of Te^0^ (0.1 mmol), at a temperature of 70 °C (±5 °C) under an inert atmosphere (N_2_). After complete reduction, the Te^2−^ solution was added to the Cd^2+^ solution, and the system heated long enough to grow the quantum dots to a desired size. Prior to application, the QDs were purified by filtration through a specific membrane (Pierce Concentrator 20 K MWCO, Thermo Scientific, Waltham, MA, USA). The pH was kept constant during all experiments (pH = 7.2).

The porphyrin ZnTE-2-PyPCl_4_ was synthesized by alkylation of the precursor Zn(II) *meso*-tetrakis (2-pyridyl)porphyrin (ZnT-2-PyP) [69] using an adaptation of a literature procedure [70]. ZnT-2-PyP (10 mg) and ethyl tosylate (400 µL = 469 mg) were dissolved in *N,N*-dimethylformamide (DMF, 2 mL) and heated to 110 °C under magnetic stirring for 24 h. The ethylation reaction was monitored by thin layer chromatography and electronic absorption spectroscopy as reported for the Mn analogue [71]. The resulting ZnTE-2-PyP^4+^ sample was isolated and purified as the chloride salt (ZnTE-2-PyPCl_4_) as described elsewhere [17,18]. Spectroscopic characterization data for ZnTE-2-PyP^4+^ (ZnP) were identical to those previously reported [17]. After synthesis, the CdTe QDs and ZnP solutions were diluted to a concentration of 10^−6^ mol·L^−1^.

### 3.2. Characterization of the Systems

The optical properties of CdTe nanocrystals and ZnP were evaluated by absorption, emission and excitation spectroscopy. The emission and excitation spectra were carried out on a PerkinElmer LS55 spectrofluorometer and the UV-Vis electronic absorption spectra were obtained using an Evolution 600 UV-Vis spectrophotometer (Thermo Scientific). The absorption spectra were also used to estimate the mean size of the QDs. The molar concentrations of the CdTe-MSA QDs suspensions were calculated by the empirical method described by Yu *et al.* [53]:

*d* = (9.8127 × 10^−7^) λ^3^ − (1.7147 × 10^−3^) λ^2^ + (1.0064) λ − 194.84 (2)

where *d* is the mean QD diameter and λ is the maximum wavelength in nm corresponding to the first excitonic absorption peak. The molar concentration was then calculated from the following equation:

*c* = *A*/(10043 × *d*^2.12^) (3)

where *d* is the mean size of a given nanocrystals sample, *A* is the absorbance of the excitonic absorption peak of the corresponding sample, *c* is the molar concentration (mol·L^−1^) of the QD suspension.

Structural characterization of CdTe nanoparticles was carried out by X-ray diffractommetry (XRD) and transmission electron microscopy (TEM). TEM was performed using a Tecnai—G2-20-FEI 2006 (Hillsboro, Oregon, USA), 200 kV, transmission electron microscope. The X-ray diffractograms (XRD) of the nanocrystals were obtained between 2θ = 15 and 60°, using an Ultima Rigako^®^, 40 kV, 20 mA current diffractometer (Washington, DC, USA), with kα_(Cu)_ = 1.544 Å radiation. The diffractograms were used to obtain information related to the structural arrangement of the material and its crystalline profile. For XRD analysis, the samples were precipitated using 99% isopropyl alcohol, at a ratio of 1:1 (QD suspension/alcohol) and centrifuged at 3600 rpm for 5 min. Thereafter, the sediment was resuspended in acetone (99%, Merck, Darmstadt, Germany), distributed on a glass plate with evaporation of acetone and analyzed by XRD.

### 3.3. Characterization of QDs-ZnP Conjugates

In order to evaluate the optical characterization and the effects on ROS generation, we chose to associate by electrostatic interactions positively charged (ZnTE-2-PyP^4+^) with negatively charged QDs (CdTe-MSA). The conjugation was performed by mixing fractions of QDs and ZnP under mechanical agitation. The molar proportions of QD and ZnP varied according to the type of test performed: (1) optical characterization and quenching evaluation [QDs-ZnP (1:0.12 to 1:1)]; (2) ROS production test [QDs-ZnP (0.12:1 to 1:1)]; (3) biological assays involving fibroblast cells and (4) *Candida albicans* [QDs-ZnP (1:1000 to 1:100)]. The chosen concentrations and molar ratios were defined according to the sensitivity of each test or based on values reported in the literature. The samples containing the mentioned mixtures of QDs and ZnP were evaluated by using absorption and emission spectroscopy and by zeta potential measurements (using a ZetaSizer Nano ZS90, Malvern, Worcestershire, UK). A preliminary study of the spectral behavior of the CdTe-MSA QDs conjugated to porphyrin molecules was performed. Aiming to evaluate a possible energy transfer of the QDs to the ZnP species during the photo-oxidation experiments, the solutions were excited at λ = 460 nm where negligible absorption of the ZnP was expected. This wavelength was determined after the optical characterization of the systems. Increasing amounts of ZnP were added to a fixed concentration of QDs maintaining a fixed volume.

### 3.4. Indirect Detection of ROS by the Oxidation of Nitrotetrazolium Blue (NBT)

One of the tests used for the determination of ROS, especially superoxide anion (O^2−^) is the known NBT oxidation technique. This method uses nitroblue tetrazolium blue chloride (NBT—Sigma-Aldrich 99%) and nicotinamide adenine dinucleotide (NADH) (Sigma-Aldrich 99%) as reagents. After addition of the photosensitizer (QDs or ZnP, or QDs-ZnP), the absorption was measured at λ = 580 nm wavelength. The evaluation of the production of ROS for the QDs, ZnP and the QDs-ZnP conjugate under irradiation of a 31 mW Light Emission Diode (LED) source at λ = 420 nm (±20 nm) was analyzed according to the increase in optical density (OD) at 580 nm as a consequence of the oxidation of the NBT solution in the presence of NADH. This wavelength was chosen after the analysis of the spectral absorbance of the isolated species and is well absorbed by both. The samples were irradiated at one-minute intervals. The amounts of each reagent used in this procedure were 80 µmol·L^−1^ (NADH), 10 mmol·L^−1^ (NBT) and 1 µmol·L^−1^ of the PS (QDs or ZnP) or molar ratios QDs-ZnP of 0.12:1 to 1:1.

### 3.5. Cytotoxicity of QDs and ZnP against Fibroblast Cells in the Presence and Absence of Radiation

We used fibroblasts to verify the cytotoxicity in mammalian cells and the possibility of using this approach, for instance, in human treatment of candidiasis. The cytotoxicity of the three samples (bare ZnP, bare QDs and conjugated samples) was initially evaluated in murine fibroblast cell lines (ATCC CRL 163) in the dark as well as after irradiation. The cells were kept in DMEM supplemented with antibiotics and antimycotics (100 units·mL^−1^ penicillin, 100 mg·mL^−1^ streptomycin, and 0.025 mg·mL^−1^ amphotericin B), 2 mmol·L^−1^ glutamine, and 10% calf serum, at 37 °C in a humidified 5% CO_2_ atmosphere until they reached 80% confluence. For subculturing and for the experiments, cells were harvested using 0.05% trypsin and 0.02% ethylenediaminetetraacetic acid (EDTA) in phosphate-buffered saline, pH 7.4. A suspension of cells was prepared at a concentration of 2 × 10^4^ per 100 μL to be seeded in each well of a 96-cell culture plate and placed in a humidified incubator for 24 h at 37 °C with 5% CO_2_ atmosphere. The culture medium was removed and replaced with 100 µL of QDs, ZnP or QDs-ZnP conjugates, which had been diluted in Gey’s buffer to a concentration range from 1 to 10 nmol·L^−1^ (QD); 10 µmol·L^−1^ (ZnP) and 1–10 nmol·L^−1^: 10 µmol.L^−1^ (QDs-ZnP). Two plates were prepared: one was exposed to light and the other was kept in the dark. The irradiated plate was kept protected from light for 10 min before being exposed to a 460 nm (±20 nm) LED source and fluence rate of 150 mW·cm^−2^ for 9 min. The non-irradiated plate had the medium replaced with the samples and treated in the same way as for the irradiated plate but without light exposure (*i.e*., the plate was left in the dark for 19 min). For both plates, the samples were then removed, the wells were washed twice with culture medium, new culture medium was added in, and the plates were returned to the incubator for 24 h. Standard solutions of 3-(4,5-dimethylthiazol-2-yl)-5-(3-carboxymethoxyphenyl)-2-(4-sulfophenyl)-2*H* tetrazolium (MTS), in the presence of phenazine methosulfate (PMS), following the MTS/PMS protocol (Promega^®^—CellTiter 96^®^ Aqueous Non-Radioactive Cell Proliferation Assay) were used to quantify the cell viability according to the equation:

(4) $$\text{CV}\left(\text{\%}\right)=\;\frac{OD_{treatment}}{OD_{control}}\;\times 100$$

where: *CV* = cell viability, *OD_treatment_* = optical density of the sample in different concentrations at 490 nm, *OD_control_* = optical density of the non-irradiated cell at 490 nm. All numerical data were pooled for each condition. Datasets were analyzed using the software Statistica 7. Comparisons were made using one-way analysis of variance using ANOVA.

### 3.6. Photodynamic Inactivation of Candida albicans

A culture of the fungus *Candida albicans* ATCC 10231 was placed in Sabouraud medium and maintained at 37 °C for 24 h to obtain a microbial culture at the stationary growth stage. The cultures were centrifuged at 2000 rpm for 10 min and resuspended in phosphate buffer saline (PBS) to a concentration of 10^7^ colony forming units (CFU)·mL^−1^. This concentration was adjusted following the method described by Kato *et al.*, using the transmittance obtained by the calibration curve at λ = 540 nm [38]. Aliquots of 180 µL of microbial suspension were transferred to a 96-well plate. Subsequently, 20 µL of photosensitizer (QDs, ZnP or QDs-ZnP) were added. The mixture was homogenized and incubated for 10 min (previously optimized incubation time). The concentrations of the QDs varied from 1 to 200 nmol·L^−1^ and the concentration of ZnP used in the experiment was fixed at 10 µmol·L^−1^, based on previous assays and data in the literature [14,72]. After an incubation time of 10 min, the samples received different doses of irradiation using a blue LED with emission at λ = 460 nm (±20 nm) with 150 mW·cm^−2^. The experiment was carried out in such a way that the spread of light in each of the irradiated wells did not interfere with the next group tested. Irradiated and non-irradiated negative control groups (without PS) were used in the experiment. CFU were counted using the method proposed by Jeff *et al*. [73]. All assays were carried out in triplicate. The LEDs used in the experiments are commercially available for routine procedures in medicine and dentistry. Datasets were analyzed using the software Statistica 7. The differences between control and treatment groups were measured using one-way analysis of variance (ANOVA) and differences with p values of less than 0.01 were considered statistically significant.

## 4. Conclusions

The results of indirect detection of reactive oxygen species show the potential of CdTe-MSA quantum dots, ZnTE-2-PyP^4+^ porphyrin and their conjugates to generate ROS, although the same behavior was not observed in the photodynamic inactivation tests involving *Candida albicans*. The CdTe QDs showed no effect in reducing cell viability of this microorganism, even after photo-activation. We believe that the PDI process was not effective on applying QDs due to the lack of uptake by the fungal cells. Moreover, in cytotoxicity assays, QDs and their conjugates were found to be more harmful against fibroblasts after light exposure. The use of isolated QDs or in combination with conventional photosensitizers for photodynamic therapy should therefore be investigated in greater depth. Conversely, the Zn(II) porphyrin proved to be promising for the photodynamic inactivation of *Candida albicans.* Detailed photophysical studies are also needed to better understand the phenomena of energy transfer between CdTe QDs and ZnP in order to better understand the photodynamic effects of these QDs- and ZnP-based PS on microorganisms and other biological systems.
